# Bone Disease in Connective Tissue Disease/Systemic Lupus Erythematosus

**DOI:** 10.1007/s00223-017-0322-z

**Published:** 2017-09-12

**Authors:** Irene E. M. Bultink

**Affiliations:** Department of Rheumatology, Amsterdam Rheumatology and immunology Center, VU University Medical Center, De Boelelaan 1117, 1081 HV Amsterdam, The Netherlands

**Keywords:** Bone mineral density, Fracture, Glucocorticoids, Osteoporosis, Systemic lupus erythematosus

## Abstract

This article reviews recent advances in the research of the mechanisms of bone loss, as well as clinical features, economic impact and therapeutic implications of osteoporosis and fractures in patients with systemic lupus erythematosus (SLE) as an illustration of bone disease in a complex systemic autoimmune connective tissue disease. Recent studies demonstrated an increased incidence of osteoporosis and peripheral and vertebral fractures in patients with SLE. The aetiology of bone loss in SLE is multifactorial, including clinical osteoporosis risk factors, systemic inflammation, serological factors, metabolic factors, hormonal factors, possibly genetic factors and medication-induced adverse effects. The incidence of symptomatic fractures in patients with SLE is increased 1.2–4.7-fold and age, disease duration, glucocorticoid use, previous cyclophosphamide use, seizures and a prior cerebrovascular event have been identified as important risk factors. Moreover, a high prevalence of morphometric vertebral fractures was demonstrated, while one in three of these patients has normal bone density, which finding points to the multifactorial aetiology of fractures in SLE. The clinical consequences and economic burden of osteoporosis and fractures as glucocorticoid treatment-related adverse events and the high frequency of glucocorticoid therapy underline the importance of reducing glucocorticoid treatment and prescribing steroid-sparing agents. No data on fall risk and its determinants and the relationship with the occurrence of fractures in patients with SLE are currently available. Fall risk might be increased in lupus patients for several reasons. In addition, the recently reported high prevalence (20%) of frailty in SLE patients may contribute to the increased fracture incidence. Therefore, the relationships between fall risk, frailty and fracture occurrence in SLE might be interesting subjects for future studies.

## Introduction

Systemic autoimmune connective tissue diseases, e.g. systemic lupus erythematosus (SLE), Sjögren’s syndrome, antiphospholipid syndrome, systemic sclerosis and idiopathic inflammatory myopathies, are chronic multifactorial disorders associated with a complex genetic predisposition and various environmental triggers inducing inflammatory and immune-mediated responses that may affect any organ system in the body. Over the last decades increasing insight is gained into alterations in bone metabolism and additional factors contributing to the increased bone loss and elevated fracture occurrence which has been demonstrated in subgroups of patients with specific systemic autoimmune connective tissue diseases. This review article focuses on SLE as an example of a systemic autoimmune disease with a complex multifactorial aetiology of increased bone loss and the occurrence of fractures.

SLE predominantly affects women (90%) and has an unpredictable course, usually characterized by alternate phases of remission and exacerbation of disease activity. The disease may involve any organ system in the body. The skin, joints, kidneys and serous membranes are frequently affected. In addition, the majority of the patients suffer from ultraviolet light intolerance and need to avoid sun exposure and to use sunscreens. Treatment of patients with SLE is directed at the suppression of disease activity and the prevention of irreversible organ damage. Frequently used agents are antimalarials (e.g. hydroxychloroquine), glucocorticoids (GCs) and immunosuppressive agents (e.g. azathioprine, mycophenolate mofetil) while severe disease complications like cerebral vasculitis or severe glomerulonephritis may need treatment with cytotoxic drugs (e.g. cyclophosphamide) or biological agents.

The far majority of patients diagnosed with SLE are relatively young women, with inherent gender-specific risks for increased bone loss. Recent studies demonstrated an increased incidence of osteoporosis [1] and symptomatic fractures [2–5] in patients with SLE compared with matched controls and provided new insights into their multifactorial aetiology. In addition, a relatively high prevalence of morphometric vertebral fractures was demonstrated in patients with SLE, while a third of these patients has normal bone density, which illustrates the multifactorial cause of fractures in SLE. The increased risk of fractures underlines the importance of prioritizing bone health as part of the treatment plan in SLE.

This review discusses recent advances in the field of the aetiology and epidemiology of osteoporosis and fractures in patients with SLE, highlighting their clinical, therapeutical and socioeconomic implications.

## Bone Loss in SLE

### Epidemiology

A recent population-based study in 7332 SLE patients and 28,079 age-matched and sex-matched controls from the United Kingdom showed a 2.53-fold increased occurrence of osteoporosis in SLE [1]. Osteopenia, defined as a T-score between −1.0 and −2.5 in the lumbar spine and/or the hip is reported in 25–74% of patients with SLE, and osteoporosis, defined as a T-score less than −2.5, in 1.4–68% of SLE patients in cohort studies [5]. These frequencies differ widely as a consequence of large differences in size, age, gender, ethnic background, disease severity and medication use between the patient groups investigated and differences in study design between studies. Most frequently, osteoporosis prevalences between 5 and 20% are reported from studies in patients with SLE.

In addition, suboptimal screening for low bone mineral density (BMD) in SLE patients is highly frequent [6, 7] and may also contribute to differences in reported frequencies of osteopenia and osteoporosis.

### Aetiology of Bone Loss in SLE

**Fig.1:**
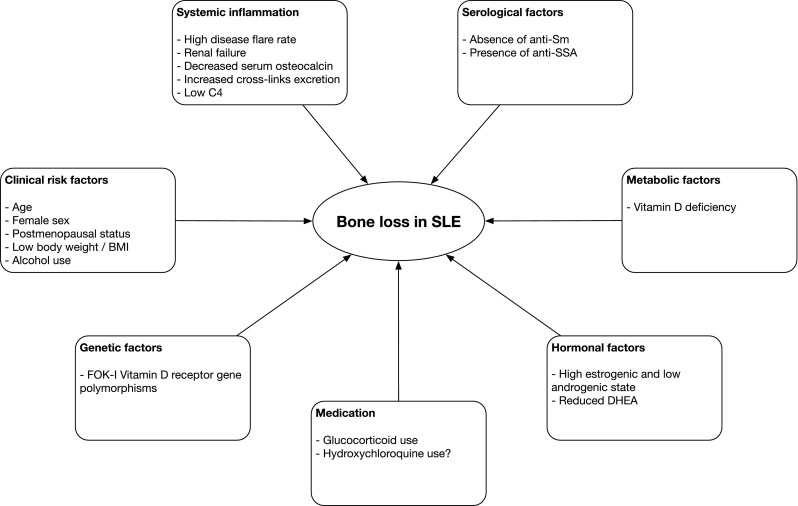
The multifactorial aetiology of bone loss in systemic lupus erythematosus. *BMI* body mass index, *DHEA* dehydroepiandrosterone, *SLE* systemic lupus erythematosus

The aetiology of the increased bone loss in SLE is supposed to be multifactorial, including clinical osteoporosis risk factors, systemic inflammation, serological factors, metabolic factors, hormonal factors, possibly genetic factors and medication-induced adverse effects (Fig. 1).

### Clinical Osteoporosis Risk Factors

Similar to findings in the general population, age [8–10], postmenopausal status [8, 11] and low body weight [8] or low body mass index [8, 9, 11] are important risk factors for osteoporosis in patients with SLE.

A recent, large population-based study in female and male patients with SLE in the United Kingdom reported gender-specific incidence rates of osteoporosis [1]. Interestingly, this study demonstrated higher absolute incidence rates of osteoporosis in female versus male SLE patients (incidence rate (IR) 12.31 per 1000 person-years (95% confidence interval (CI) 11.22–13.50) versus IR 6.22 (95% CI 4.56–8.47), but men with SLE had a higher relative risk of osteoporosis than women (incidence rate ratio (IRR) 5.35 (95% CI 3.41–8.40) in male SLE patients versus 2.41 (95% CI 2.16–2.70) in female patients.

In the general population, white ethnicity is associated with an increased osteoporosis risk, whereas African American ethnicity is associated with a higher peak bone mass [12]. However, studies on the influence of ethnic background on bone mass in SLE show conflicting results. Two studies in SLE patients showed an association between white [13] or non-African Caribbean ethnicity [14] and reduced bone mass. But, another study demonstrated reduced BMD in African American females with SLE as compared to whites, after controlling for clinical variables and after adjustment for glucocorticoid (GC) use [15].

Smoking and excessive alcohol use have adverse effects on bone mass in the general population. However, smoking has not been reported as an independent risk factor for osteoporosis in several studies in patients with SLE [9, 11, 14, 16, 17]. Alcohol use was associated with reduced bone mass in one study in male SLE patients from Hong Kong [16].

### Role of Systemic Inflammation

Systemic inflammation in SLE may contribute to bone loss by increasing osteoclastic bone resorption and by reducing osteoblastic bone formation. In patients with active disease, increased serum levels of tumor necrosis factor (TNF) [18] and elevated levels of oxidized low-density lipoprotein (LDL) [19] were shown. Oxidized lipids can activate T cells, which may induce increased production of receptor activator of nuclear factor-κB (RANKL) and TNF. Both RANKL and TNF stimulate osteoclast differentiation and activity [18]. Moreover, oxidized LDL might also negatively influence bone formation by reducing osteoblast maturation [20].

A study in premenopausal women with untreated and recently diagnosed SLE demonstrated decreased serum levels of osteocalcin, a marker for bone formation, and increased cross-links excretion in the urine, a marker for bone resorption [21], which finding points to a change in bone metabolism due to the disease itself. A study in recently diagnosed male lupus patients from Hong Kong showed decreased osteocalcin serum levels in SLE patients versus controls and also demonstrated a negative association between osteocalcin serum levels and disease activity [22]. Moreover, low complement C4 levels (a measure of active disease) were a predictor of low BMD in the lumbar spine in the Hopkins Lupus Cohort [8]. Since low C4 is a marker of disease activity in SLE, this finding points to a negative effect of systemic inflammation, due to active disease, on bone mass in SLE.

Several cross-sectional studies failed to show a relationship between disease activity and BMD in SLE [9, 11, 13, 23], which finding might be explained by the cross-sectional design of those studies: the influence of disease activity on bone mass in a disease with a fluctuating disease course characterized by exacerbations and remissions is supposed to be assessed better by using multiple measurements of disease activity over time or by measuring the frequency of disease flares over time in relationship to change in BMD. A recent 5-year follow-up study in Chinese women with SLE indeed demonstrated an association between high rate of disease flares and increased bone loss in SLE [24], which finding confirms the hypothesis that systemic inflammation contributes to bone loss in SLE.

Inflammation-induced lupus nephritis occurs in up to 60% of the SLE patients ever during the disease course and can result in renal failure [25]. In severe renal failure, bone loss is increased by the development of secondary hyperparathyroidism, increased osteoclastic bone resorption and by the development of low 1,25-dihydroxy vitamin D serum levels, which might impair intestinal calcium absorption. However, an association between impaired renal function and low bone mass was reported from only one study, in older lupus patients [9]. Importantly, the majority of studies performed employed reduced renal function as an exclusion criterion. Therefore, the influence of renal failure on bone loss in SLE is still partly unclear.

### Serological Factors

Data on the role of autoantibodies in regulating bone metabolism in SLE are very scarce. In a cross-sectional study in 34 postmenopausal Chinese women with SLE, an association between the presence of anti-Sm and higher BMD of the hip was demonstrated, whereas the presence of anti-Ro was negatively associated with hip bone mass [26]. The relationship between the presence of anti-Ro and lower bone mass may be explained by the fact that anti-Ro positive SLE patients are usually advised against sun exposure [26]. No relationship between the presence or absence of anti-dsDNA antibodies and bone mass in SLE was found [26].

The role of other autoantibodies in regulating bone mass in SLE is unknown. The presence of antibodies against citrullinated protein (ACPA) has been associated with significant bone loss in healthy persons, which finding is explained by the fact that ACPA directly stimulates osteoclast differentiation which induces bone loss [27]. ACPA might also contribute to increased bone loss in the subgroup of ACPA positive SLE patients, but the contribution of ACPA to reduced BMD in the majority of SLE patients is probably limited since the prevalence of ACPA positivity is much lower in patients with SLE compared to patients with rheumatoid arthritis.

### Metabolic Factors

An increased prevalence of vitamin D deficiency, a metabolic condition that may induce bone loss, has been demonstrated in SLE patients from different geographical regions [28–30]. A cross-sectional study in Dutch SLE patients demonstrated low 25-hydroxy vitamin D (25(OH)D) serum levels were significantly associated with low spine BMD [11]. A 6-year prospective study in 126 Dutch lupus patients showed that low 25(OH)D serum levels at baseline were independently associated with bone loss in lumbar spine and hip [31].

Several factors may contribute to vitamin D deficiency in patients with SLE: photosensitivity and use of sunscreens, dark skin pigment, renal failure, disease activity, GC therapy and anti-vitamin D antibodies.

Ultraviolet light intolerance is present in the majority of SLE patients and leads to avoidance of sun exposure and subsequently reduced de novo vitamin D synthesis in the skin. Lupus patients with dark skin pigment may be at increased risk for vitamin D deficiency because melanin blocks vitamin D synthesis in the skin. A study from the United States showed reduced vitamin D serum levels in African American SLE patients compared to white patients [32]. Moreover, patients with SLE are in general advised to use sunscreens continuously, which may lead to decreased or even completely stopped vitamin D synthesis in the skin and subsequently reduced vitamin D serum levels [33].

Patients with SLE may develop renal failure as a consequence of lupus-related renal inflammation and/or hypertension, which may induce reduced 1,25(OH)_2_D levels. A study in Canadian SLE patients indeed demonstrated a relationship between high serum creatinine levels and low 1,25(OH)_2_D levels [30].

The relationship between disease activity and vitamin D status in SLE is not fully clear. In contrast to cross-sectional studies reporting a negative association between lupus disease activity and vitamin D levels, a large longitudinal study in over 1000 patients from the Hopkins Lupus Cohort [34] demonstrated that a 20 ng/ml increase in 25(OH)D level in lupus patients with low 25(OH)D serum levels (<40 ng/ml) who received vitamin D supplementation was associated with a 21% decrease in the odds of having a high disease activity score. Although this association was statistically significant, the clinical importance is modest.

Chronic GC therapy has been demonstrated to change vitamin D metabolism, which leads to the formation of more biologically inactive metabolites, resulting in reduced intestinal calcium absorption [35]. A study in patients with SLE showed an association between cumulative GC use and low levels of 25(OH)D and 1,25(OH)_2_D [30]. The antimalarial hydroxychloroquine (HCQ) is frequently used for the treatment of SLE and is supposed to inhibit the conversion of 25(OH)D in 1,25(OH)_2_D by inhibiting the enzyme hydroxylase α1. However, studies on the influence of HCQ on vitamin D status in SLE patients showed conflicting results [13, 28, 36].

Anti-vitamin D antibodies have been detected in 4% of SLE patients, but their clinical significance is unclear since the presence of these antibodies was not related to vitamin D levels [37].

### Hormonal Factors

Sexual hormones have a protective effect on bone mass. Usually, patients with SLE have high oestrogen levels, but reduced androgen levels compared to age- and sex-matched subjects. Levels of dehydroepiandrosterone (DHEA), a measure of the adrenal androgen status, are usually decreased in patients with SLE compared to age- and sex-matched controls and patients with active disease have lower levels than those with quiescent disease [38]. In the general population, serum DHEA levels progressively decline with age from the third decade on, and this decrease is associated with age-related bone loss. In patients with SLE, the low androgenic state seems to influence bone metabolism stronger than the relatively high oestrogen levels. A study in premenopausal women with SLE demonstrated an association between low DHEA sulphate levels and low BMD [39], which offers the opportunity for the therapeutic use of DHEA to prevent bone loss in patients with SLE.

### Genetic Factors

FOK-I vitamin D receptor (VDR) gene polymorphisms may influence bone mass in SLE. A study in Dutch SLE patients demonstrated significantly higher mean BMD values in the lumbar spine in patients carrying the ff genotype of the FOK-I VDR compared to patients with the FF and Ff genotypes [40]. This finding might be in part explained by the association found between FOK-I VDR gene polymorphisms and vitamin D status in SLE: patients carrying the FOK-I ff genotype have increased 25(OH)D serum levels compared to patients with the FF genotype [41]. However, no association between FOK-I VDR gene polymorphisms and BMD at the hip was found, and average BMD changes in both lumbar spine and hip were not significantly different between the three genotypic groups during 5.3 years of mean follow-up [40].

### Role of Medication

GCs are frequently used for the treatment of SLE disease flares and complications. GCs play a dual role with respect to bone mass: these drugs are wellknown to induce bone loss, but might also have beneficial effects on bone mass by reducing the adverse effects of systemic inflammation on bone. Several cross-sectional studies assessed the influence of GC use on bone mass and demonstrated conflicting results. Longitudinal follow-up studies on this subject are scarce, but two larger prospective studies [24, 31] demonstrated that bone loss occurs predominantly in SLE patients treated with at least 7.5 mg prednisone per day, whereas use of a lower daily prednisone dosage is not associated with bone loss.

The influence of HCQ treatment on bone mass in SLE is not clear. HCQ use was associated with higher BMD in the lumbar spine [13, 26] and at the hip [13] in two cross-sectional studies in female lupus patients. But, a cross-sectional study in male SLE patients reported significantly lower BMD in the spine and hip in patients who ever used HCQ compared to patients who had never taken HCQ [16]. However, in that study, HCQ users more frequently suffered chronic arthritis and photosensitivity and they were more frequently smokers compared to non-users, which might be confounding variables regarding the negative association found between HCQ use and BMD. The 6-year follow-up study in Dutch patients with SLE showed significant reduction in hip bone mass in patients using HCQ [31], but the 5-year prospective study in Chinese lupus patients did not demonstrate an influence of HCQ use on bone mass [24]. Further research on this subject is important, since the majority of SLE patients is currently treated with antimalarials, since these agents are regarded as ‘anchor drugs’ for the treatment of SLE.

The associations found between low body weight/low body mass index, low 25(OH)D serum levels, systemic inflammation and treatment with higher dosages of GCs and increased bone loss in patients with SLE are important for clinical practice. Physicians should give advice for maintaining normal body weight and prescribe vitamin D supplementation. In addition, treatment of systemic inflammation with immunosuppressive medication is of major importance, not only to reduce disease activity (since occurrence of disease flares has been associated with increased bone loss in SLE), but also to reduce dosage and duration of GC therapy in patients with frequent disease flares or chronic active disease who are frequently or chronically treated with GCs. Determination of a ‘safe’ GC dose for chronic daily use is of major importance and should be subject of future studies in large patient populations. Moreover, future research should also focus on the influence of gender and ethnic background on bone mass, and on the influence of autoantibodies and hydroxychloroquine use on bone metabolism in SLE.

Table 1 summarizes the (possible) risk factors for bone loss in SLE and their relevance.

**Table 1:** Possible risk factors for increased bone loss in patients with systemic lupus erythematosus

Possible risk factor	Independent risk factor for increased bone loss?	Relevance	References
Clinical risk factors			
Age	Yes	+++	[8–10]
Female sex	No; absolute risk higher in females but relative risk higher in males	+	[1]
Postmenopausal status	Yes	+++	[8, 11]
Low body weight/low BMI	Yes	+++	[8, 9, 11]
Ethnicity	Unclear	±	[13–15]
Alcohol use	Yes	+	[16]
Smoking	No	+++	[9, 11, 14, 16, 17]
Systemic inflammation			
Low C4 serum levels	Yes	+	[8]
High disease flare rate	Yes	++	[24]
Renal failure	Yes	+	[9]
Serological factors			
Absence of anti-Sm	Yes	+	[26]
Presence of anti-Ro	Yes	+	[26]
Metabolic factors			
Low 25(OH)D serum levels	Yes	++	[31]
Hormonal factors			
Low DHEA serum levels	Yes	+	[38]
Genetic factors			
FOK-I VDR FF or Ff genotype	Yes	+	[40]
Medication			
Glucocorticoid use ≥ 7.5 mg daily	Yes	++	[24, 31]
HCQ use	Unclear	±	[13, 16, 24, 26, 31]

Classification of relevance: + = limited evidence; ++ = clear evidence from prospective study/studies; +++ = strong evidence from three or more studies; ± = unclear due to conflicting results from studies

*BMI* body mass index, *25(OH)D* 25-hydroxy vitamin D, *DHEA* dehydroepiandrosterone, *VDR* vitamin D receptor, *HCQ* hydroxychloroquine

## Fractures in SLE

### Epidemiology and Aetiology of Symptomatic Fractures

Only a few population-based studies on all symptomatic fractures in SLE were performed and demonstrated a 1.2–4.7-fold increased incidence of all symptomatic fractures in SLE patients versus sex-matched and age-matched healthy controls [2–4, 42] (Table 2). Three of these studies [2–4] were performed in populations from specialized tertiary lupus referral centres in university hospitals and reported the highest relative risks. However, investigating fracture incidence with data from a tertiary referral centre may lead to an overestimation of fracture occurrence due to the selection of patients with a more severe disease course. The largest population-based study [42] on all symptomatic fractures was performed in the United Kingdom and used data from the Clinical Practice Research Datalink, a general practitioners database. This study in 4343 SLE patients and 21,780 matched controls showed a slightly increased relative risk of symptomatic fractures (RR 1.22, 95% CI 1.05–1.42) in lupus patients compared to healthy controls. A systematic review and meta-analysis comprising population-based studies on fractures in SLE confirmed an increased fracture occurrence at all locations, before and after adjusting for confounding variables (unadjusted RR 2.07, 95% CI 1.46–2.94, *P* < 0.001; adjusted RR 1.22, 95% CI 1.05–1.42, *P* = 0.01) [43]. Subgroup analyses by fracture types showed that SLE was significantly associated with increased risks for hip fracture and vertebral fracture in patients of both sexes [43].

**Table 2:** Population-based studies on symptomatic fractures in patients with systemic lupus erythematosus

Authors, year	Country	Setting	Fracture types	Number of patients	Number of controls	Female gender (%)	Mean age (years)	Mean ± SD disease duration (years)	Follow-up duration (years)	RR (95% CI)	Risk factors
Ramsey-Goldman et al, 1999 [2]	USA	University hospital	All	702	89,100	100	45.4	11.0 ± 7.1	8.2	4.70 (3.80–5.80)	Older age at lupus diagnosis Duration of GC use
Rhew et al, 2008 [3]	USA	University hospital	All	100	100	100	44.1	8.8 ± 8.3	2	3.30 (1.10–10.00)	Alcohol use
Weiss et al, 2010 [50]	Sweden	National hospital discharge register (SNHDR)	All	136	952	Not reported	Not reported	Not reported	Not reported	2.90 (2.20–3.90)	Not reported
Ekblom-Kullberg et al, 2013 [4]	Finland	University hospital	All	222	720	92	47.0	13.1 ± 9.8	13.1	1.80 (1.30–2.40)	Age Duration of GC use Comorbidity
Wang et al, 2013 [44]	Taiwan	Nationwide	Hip only	14,544	14,544	90	38.1	6.0 ± 2.2	6.0	3.17 (1.92–5.39) femoral neck 1.11 (0.58–2.11) trochanteric	Age Seizures Past cerebrovascular event Previous IV cyclophosphamide use
Bultink et al, 2014 [42]	United Kingdom	General practitioners database (CPRD)	All	4,343	21,780	89	46.7	6.4 ± 5.1	6.4	1.22 (1.05–1.42)	GC use in previous 6 months Disease duration Seizures Past cerebrovascular event Previous fracture

*SD* standard deviation, *RR* relative risk, *CI* confidence interval, *GC* glucocorticoids, *SNHDR* Swedish National Hospital Discharge Register, *IV* Intravenous, *CPRD* Clinical Practice Research Datalink

The study from the United Kingdom [42] revealed that SLE patients with seizures or a history of stroke have a high risk for symptomatic fractures. A nationwide study on hip fractures in lupus patients from Taiwan [44] confirmed an increased risk of femoral neck fractures (hazard ratio 2.29, 95% CI 1.02–5.15) in SLE patients with a history of stroke compared to patients without stroke. The increased fracture occurrence in patients with seizures or a past cerebrovascular event might be explained by an increased fall risk in these subgroups of patients and by adverse effects of antiepileptic drugs and anticoagulants on bone mass. Indeed, a recent cohort study in Italian SLE patients [17] demonstrated both use of antiepileptic drugs and use of anticoagulants as independent risk factors for symptomatic fractures. The nationwide study from Taiwan [44] reported previous intravenous cyclophosphamide use as an independent risk factor for femoral neck fractures in patients with SLE. This finding might be explained by cyclophosphamide-induced premature ovarian failure inducing reduced bone mass and an increased fracture risk. However, this association may also be related to an increased occurrence of fractures in SLE patients with severe disease complications, since cyclophosphamide pulse therapy is usually prescribed to patients with severe disease manifestations, e.g. cerebral vasculitis or lupus nephritis.

**Fig. 2:**
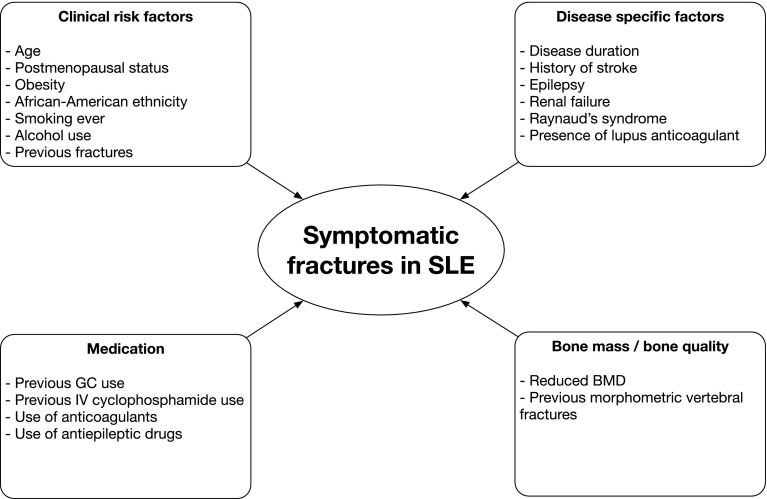
The multifactorial aetiology of symptomatic fractures in patients with systemic lupus erythematosus. *BMD* bone mineral density, *GC* glucocorticoid, *IV* intravenous, *SLE* systemic lupus erythematosus)

Cohort studies in patients with SLE have reported the occurrence of symptomatic fractures since lupus diagnosis in 4.4–18.8% of patients [2, 8, 11, 14, 17, 45–49] and the hip, vertebra, rib, foot, ankle and arm are the most frequent locations of fractures [2, 8, 17, 45, 47]. Population-based studies identified age [4], disease duration [2, 42], alcohol use [3], seizures [42], history of stroke [42], previous GC use [2, 4, 42] and previous cyclophosphamide pulse therapy [44] as risk factors for symptomatic fractures. In addition, African American ethnicity [48], postmenopausal status [8], obesity [48], smoking [48], renal failure [48], Raynaud’s syndrome [48], presence of lupus anticoagulant [48], reduced BMD [14, 45, 49], use of anticoagulants [17] and use of antiepileptic agents [17] are identified as risk factors for fractures from cohort studies (Fig. 2).

### Epidemiology and Aetiology of Vertebral Fractures

Only two population-based studies on fractures in SLE reported data on symptomatic vertebral fractures. A study [50] from Sweden on fracture occurrence in patients with rheumatic diseases, including 136 patients with SLE, showed an increased risk [odds ratio (OR) 2.2] for symptomatic vertebral fractures in SLE patients versus age-matched and sex-matched healthy controls. A study from Finland [4] reported a 4-fold increased risk (OR 4.0, 95% CI 1.8–8.6) for symptomatic vertebral fractures in 204 women with SLE compared to controls. Importantly, both studies reported unadjusted risk rates only. A meta-analysis, which included both studies, demonstrated the relative risk of symptomatic vertebral fractures was almost 3-fold increased (RR 2.97, 95% CI 1.71–5.16, *P* < 0.001) in SLE patients versus healthy subjects [43].

However, studies focusing on symptomatic vertebral fractures only have a disadvantage, since studies in the general population [51] and in SLE [11, 45] have shown that the majority of vertebral fractures do not come to clinical attention. The identification of prevalent morphometric vertebral deformities is important, as these vertebral fractures are associated with a reduced quality of life [46], an increased risk for future major osteoporotic fractures and hip fractures in patients with SLE [46] and an increased mortality risk in the general population [52].

Eight cross-sectional studies [11, 46, 53–58] and two longitudinal studies [45, 59] on the prevalence of vertebral fractures, which all assessed vertebral deformities using the standardized semiquantitative method of scoring vertebral deformities by Genant [60], have been published (Table 3). These studies demonstrated the presence of at least one vertebral fracture in 13.7–50% of SLE patients, despite relative young mean age (32–48 years). The majority of (larger) studies [11, 45, 46, 53–57, 59] showed vertebral fracture prevalence rates of 13.7–29%, while a small study in 52 Japanese lupus patients revealed a remarkably high prevalence (50%) of vertebral fractures [58].

**Table 3:** Studies on prevalent morphometric vertebral fractures in patients with systemic lupus erythematosus

Authors, year	Study design	Country	Number of patients	Mean age ± SD (years)	% with ≥ 1 vertebral fracture	Risk factors
Bultink et al [11]	Cross-sectional	Netherlands	90	41 ± 13	20	IV methylprednisolone Male gender
Borba et al. [53]	Cross-sectional	Brazil	70	32 ± 8	21.4	Low BMD associated with number of fractures
Mendoza-Pinto et al. [54]	Cross-sectional	Mexico	210	43**	26.1	Age Low hip BMD
Li et al. [55]	Cross-sectional	Hong Kong	152	48 ± 10	20.4	Age High BMI Low spine BMD
Almehed et al. [56]	Cross-sectional	Sweden	150	47 (20–82)*	29	Age Low hip BMD
Garcia-Carrasco et al. [57]	Cross-sectional	Mexico	140	43 (18–76)*	24.8	N.A.
Furukawa et al. [58]	Cross-sectional	Japan	52	45 ± 13	50	Previous fractures
Zhu et al. [45]	Longitudinal	Hong Kong	127	47 ± 10	18.1	N.A.
Rentero et al. [46]	Cross-sectional	Spain	95	45 ± 14	13.7	N.A.
Garcia-Carrasco et al. [59]	Longitudinal	Mexico	110	42 ± 11	20	Low hip BMD Disease duration

*Values represent median (range); ** Value represents median, range not reported

*SD* standard deviation, *BMD* bone mineral density, *BMI* body mass index, *IV* intravenous, *NA* not assessed

As expected, the majority of vertebral fractures are mild deformities and are located in the thoracic spine [11, 46, 53–59]. Age [46, 54–56], duration of menopause [46], high body mass index [55], male sex [11], previous use of methylprednisolone pulse therapy [11], previous fractures [58] and low BMD [53–56] have been associated with prevalent vertebral fractures in SLE.

Incidence rates of vertebral fractures in SLE were reported from two prospective cohort studies. A recent 8-year follow-up study [59] in 110 female SLE patients from Mexico with mean age 42.3 years demonstrated at least one new morphometric vertebral fracture in 32% of the patients during follow-up, while 20% of the patients had a morphometric vertebral fracture at baseline, which resulted in an annual incidence rate of new morphometric fractures of 3.5 (95% CI 2.40–4.91) per 100 patient years. The annual incidence rate reported from this study is much higher than the annual incidence rate (1.07 per 100 patient years) of morphometric vertebral deformities in females (mean age 62.2 years) from the general population as demonstrated in the European Prospective Osteoporosis Study (EPOS) [61].

In the study on Mexican female lupus patients [59], longer disease duration and low hip BMD were identified as independent risk factors for new morphometric vertebral deformities. Surprisingly, the majority of new vertebral deformities occurred in the lumbar spine, whereas most prevalent fractures were located in the mid-thoracic spine and thoracolumbar region of the spine. In contrast to the high prevalence of vertebral fractures demonstrated in cross-sectional studies and the relatively high annual incidence rate of new vertebral fractures as reported from the study in Mexican SLE patients, a 5-year prospective study in 127 Chinese women with SLE [45] reported a very low annual incidence rate (0.94 per 100 patient years) of vertebral fractures, while 18.1% of the patients had at least one vertebral deformity at baseline. Osteoporosis of the lumbar spine was identified as the most important risk factor for incident vertebral fractures. Remarkably, in the study by Zhu and colleagues, the majority of incident vertebral fractures were classified as severe (grade III, >40% reduction in vertebral height) deformities, which is in sharp contrast with the mild (grade I) prevalent deformities in cross-sectional studies and the mild-to-moderate incident vertebral fractures reported from the Mexican study. The unexpected severity of incident vertebral fractures found in the study by Zhu and colleagues suggests the inclusion of traumatic fractures. Besides low spine BMD, Zhu and colleagues [45] also reported higher organ damage score (SLICC/ACR damage index) as a predictor of new vertebral fractures. However, the SLICC/ACR damage index comprises osteoporotic fractures as a damage item and, therefore, the authors should have used a modified SDI score, which excludes osteoporotic fractures as a damage item for the assessment of risk factors for incident vertebral fractures.

The limited number of studies on incident vertebral fractures and their conflicting results illustrate the need for further studies in large groups of patients with SLE to establish a more precise estimation of vertebral fracture incidence and the associated risk factors.

Despite the fact that the majority of vertebral fractures occur asymptomatic, the detection of vertebral fractures is not only important because of their clinical implications in terms of reduction in quality of life and an increased risk of future fractures, but vertebral fractures may also have therapeutic consequences. According to the recent National Osteoporosis Foundation [62] and the National Osteoporosis Guideline Group [63] guidelines, the detection of one or more asymptomatic vertebral fractures in patients with a BMD in the osteopenic range is an indication for initiation of anti-osteoporosis agents. In line with these guidelines, the assessment of vertebral deformities in addition to bone density measurement is recommended in the management of all SLE patients in whom bone density measurement is indicated because of the presence of clinical risk factors for fractures [5].

## Relationship Between Bone Mineral Density and Fractures in SLE

Although an increased prevalence of reduced BMD and an increased incidence of fractures have been demonstrated in patients with SLE, the relationship between bone density and fracture occurrence in SLE is complicated and not yet fully clear. Some cohort studies [8, 14, 45] reported reduced BMD as a significant risk factor for symptomatic fractures, and a significant association between low BMD in the lumbar spine or at the hip and prevalent morphometric vertebral deformities [53–56]. In addition, a recent 8-year prospective study [59] in Mexican women with SLE reported low hip BMD as an independent risk factor for new morphometric vertebral deformities. However, other studies [11, 17, 58] did not demonstrate a relationship between bone density and vertebral fractures in lupus patients. In addition, two studies [54, 55] demonstrated normal BMD in 29-35% of the SLE patients with one or more vertebral fractures.

These discrepancies might be explained by the limited value of BMD measurement in the assessment of fracture risk and the multifactorial cause of fractures in SLE. The assessment of BMD using dual energy X-ray absorptiometry (DXA) measurement implicates measurement of the total amount of calcium/hydroxyapatite in a specific body region, which method of measurement may be influenced by the presence of atherosclerosis or spondyloarthritis. In addition, DXA does not comprise assessment of connectivity of trabeculae and assessment of other aspects of bone quality.

High-resolution peripheral quantitative computed tomography (HR-pQCT) is an emerging method for the assessment of bone microarchitecture, geometry and bone strength. A limited number of studies using HR-pQCT as imaging modality in SLE were published and demonstrated reduced bone strength in patients with SLE (both in patients on long-term GC therapy and in patients not using GCs) compared to healthy controls [64, 65]. HR-pQCT might be a better modality for the prediction of fracture risk in patients with SLE than DXA. However, this technique is currently not recommended as a screening method since it is costly, not routinely available in clinical practice, and the predictive value of HR-pQCT for fracture occurrence in SLE patients has not been proven yet.

The occurrence of vertebral fractures in SLE patients with normal or only slightly reduced BMD also illustrates the multifactorial aetiology of fractures in a heterogenous and complex disease as SLE. Besides clinical risk factors as age, postmenopausal status, ethnic background and previous fractures, treatment-related factors and specific disease-related factors are supposed to be important additional factors contributing to fracture risk in SLE.

Treatment with GCs is regarded as an important additional risk factor for fractures in SLE, since patients with SLE are frequently treated with GCs and GC use has been associated with an increased fracture risk in individuals as compared to persons who have similar BMD values but do not use GCs [66]. In three out of four population-based studies in patients with SLE [2, 4, 42] previous use of these agents was identified as an important and independent risk factor for the occurrence of fractures. In addition, a study [67] in the Hopkins Lupus Cohort demonstrated a dose-dependent effect of GC use on fracture risk: exposure to a mean prednisone dose of 7.5 mg/day or higher in the past versus less than 7.5 mg/day increased the risk of osteoporotic fractures (HR 2.16, *P* < 0.001). This finding was confirmed by a study [68] in Spanish patients with SLE.

Patients with neurological disease complications, especially a past stroke [17, 42] or epilepsy [17] have been identified at high risk for fractures, which findings might be related to an increased fall risk. No data on fall risk, frequency of falls and the relationship with the occurrence of fractures in patients with SLE are currently available, but fall risk in SLE may be increased due to arthritis, fatigue, neurological complications, visual impairment or muscle weakness (which might be related to GC use, inactivity and vitamin D deficiency). A study [69] showed a relationship between reduced lower extremity muscle strength and physical disability in patients with SLE, which might be related to an increased risk of falls and fractures. In addition, a recent study [70] in 152 women with lupus with mean age 48 years reported a high prevalence (20.4%) of frailty, a rate twice as high as that seen in persons from the general population aged 65 years and older [71]. A systematic review of studies involving community-dwelling older people showed that frailty is a significant predictor of future fractures [72], which finding suggests that the high prevalence of frailty in lupus patients may be an additional factor raising fracture risk in patients with SLE.

## Economic Burden of Osteoporosis and Fractures in SLE

The economic impact of osteoporosis and fractures in patients with SLE has recently been evaluated by a Delphi panel of rheumatologists from the United States, who are experts in the treatment of SLE [73]. This panel reported that osteoporosis is the most frequently occurring GC-related adverse event with an estimated frequency of 10–20% in SLE patients using GC dosages <20 mg daily and an estimated prevalence of 21–50% in patients using GC dosages >20 mg daily. Importantly, 35–50% of patients with mild SLE, more than 90% of patients with moderate SLE and more than 95% of patients with a severe disease course are treated with GCs. The total cost of treatment of osteoporosis in a lupus patient in the United States was estimated $4170, which amount includes specialist consultations and BMD measurement but excludes medication [73]. This study also demonstrated that fractures were one of the three GC-related adverse events in SLE patients with the highest estimated total non-pharmacological costs per event: $8220 [73]. The results of this study not only demonstrate the economic impact of osteoporosis and fractures, but also illustrate the importance of reducing dosages and duration of GC therapy and the importance of prescribing steroid-sparing agents in patients with SLE.

## Prevention and Treatment of Osteoporosis and Fractures in SLE

### General Measures

Lifestyle measures are important for the prevention and treatment of osteoporosis and fractures. These include avoidance of smoking, limiting alcohol use, maintaining a normal body weight, avoiding falling and regularly performing weight-bearing physical activity [74]. However, to date, no evidence from randomized controlled trials has been obtained yet that modification of lifestyle is associated with a significant positive effect on bone mass and/or a reduction in fracture occurrence in patients with SLE.

Extra focus should be put on an adequate calcium and vitamin D intake [74], which are important for bone mineralization and neuromuscular function. The total calcium intake (dietary intake plus supplementation) should be at least 1000 mg per day, while in GC users 1200–1500 mg/day is recommended [75]. Vitamin D supplementation (800–1000 IU/day) is recommended in patients with insufficient 25(OH)D serum levels, which is highly frequent in SLE patients, and in patients using GCs [75].

Treatment with immunosuppressive medication is of major importance, not only to reduce disease activity-related inflammation-induced bone loss, but also to minimize dosage and duration of GC therapy.

### Assessment of Fracture Risk

The aetiology of osteoporotic fractures is multifactorial, including bone-related and fall-related factors. Several algorithms for calculating the absolute fracture risk for an individual patient have been developed, of which the Fracture Risk Assessment (FRAX) tool as proposed by the World Health Organization [76] is a frequently used model. The FRAX instrument can be used to calculate the 10-year risk for both hip fracture and major osteoporotic fracture (hip, proximal humerus, wrist and clinical vertebral fracture). Unfortunately, there is lack of consensus regarding at which cutoff point treatment with anti-osteoporosis medication should be initiated, but the National Osteoporosis Foundation of the United States advises to start treatment in individuals with 10-year risk FRAX scores of 3% or more for hip fracture and/or a 20% or more FRAX risk score for major osteoporotic fracture [77]. Using the FRAX instrument to calculate fracture risk in patients with SLE has disadvantages since the FRAX tool takes into account BMD and family history, but does not comprise the evaluation of risk factors for falls and the presence of prevalent vertebral deformities, while prevalent vertebral fractures are frequently observed in SLE patients. In addition, the FRAX tool takes GC use into account, but not (cumulative) GC dose. Furthermore, the FRAX instrument was developed for postmenopausal women only and is invalid in individuals priorly treated with bisphosphonates.

As a consequence of the above-mentioned reasons, FRAX might underestimate fracture risk in SLE. Two studies using the FRAX instrument in patients with SLE were published and reported relatively high estimated 10-year probability rates of hip and major fractures in lupus patients. A study in 271 Canadian women with SLE [78] without prior osteoporotic fractures demonstrated a 10-year probability of a hip fracture (FRAX-hip) ≥3% in 9.4% of the patients and a 10-year probability of a major fracture (FRAX-major) ≥20% was found in 5.3% of the patients. A study [79] in 45 patients with SLE from Singapore and 45 healthy subjects reported significantly higher estimated 10-year probability of hip and major fractures in SLE patients compared to healthy controls, while BMD values of the hip and lumbar spine were comparable. The FRAX tool is not recommended for calculating fracture risk in all SLE patients in daily clinical practice because of the many disadvantages mentioned. In addition, none of the many other fracture risk assessment tools has been validated for use in SLE patients.

### Drug Treatment

For SLE patients with osteoporosis, with a previous fragility fracture and/or those receiving GC therapy, it is of major importance to consider the prescription of anti-osteoporosis drugs.

Bisphosphonates are the mainstay of osteoporosis treatment for postmenopausal women and for individuals with GC-induced osteoporosis without renal impairment. A meta-analysis of bisphosphonate therapy in patients with rheumatic diseases treated with GCs demonstrated decreased fracture incidence and improvement in BMD in both prevention and treatment intervention studies [80]. In patients with SLE using GCs, alendronate [81], pamidronate [82] and ibandronate [83] have been demonstrated to improve BMD. In patients with GC-induced osteoporosis, treatment with zoledronic acid once yearly intravenously has been proven superior to other bisphosphonates with respect to increase in BMD [84]. Bisphosphonate use in premenopausal women is a point of discussion. Use of these agents has been associated with foetal abnormalities in animal studies [85]. However, the teratogenic effects of bisphosphonates in humans are disputed, since human preconception and first trimester bisphosphonate use has to date not been associated with the same adverse effects as reported from animal studies [86]. Large, prospective studies in premenopausal women with preconception and perinatal bisphosphonate exposure are needed to definitely assess the safety of these agents during pregnancy.

In addition, physicians should remind their patients that osteonecrosis of the jaw [87] and atypical femoral shaft fractures [88] are rare but potential complications of bisphosphonate treatment.

The anabolic agent teriparatide increases bone formation and bone strength through inducing osteoblast formation and reducing apoptosis. Teriparatide has been demonstrated to be superior to bisphosphonates in patients with GC-induced osteoporosis in increasing BMD and in reducing incidence of vertebral and non-vertebral (but not hip) fractures [89]. PTH analogues are mainly used for the treatment of patients with severe osteoporosis in whom treatment with bisphosphonates has failed or is not tolerated, because of the high cost of these drugs.

Denosumab, a monoclonal antibody against RANKL, acts through decreasing osteoclast differentiation, survival and activity, which results in decreased bone resorption. A recent study has demonstrated superiority to bisphosphonates with respect to increase in BMD in chronic GC users, although this study did not use fractures as endpoint [90]. This drug is attractive as a therapeutic agent for the subgroup of lupus patients with impaired renal function and might be attractive for the treatment of premenopausal women since denosumab is not incorporated in bone. During denosumab treatment serum calcium levels should be monitored since hypocalcemia is an important possible side-effect of this agent [91]. In addition, an increased risk for atypical femoral shaft fractures [88] and osteonecrosis of the jaw [92] have been reported as adverse effects of denosumab. Moreover, physicians should be aware that discontinuation of denosumab treatment has been associated with the occurrence of severe rebound vertebral fractures [93].

The use of oestrogen-containing drugs to prevent bone loss in SLE is not recommended since these agents are associated with an increased risk of thrombotic events in the general population and an increased flare rate in patients with SLE [94], although only the incidence of mild-to-moderate flares was increased in this study, not severe flares. HRT users suffered more thrombotic events than non-users, but this difference was not statistically significant. However, SLE patients with prior thrombosis, high titers of anticardiolipin antibodies or presence of lupus anticoagulant were excluded from this study. The oestrogen receptor modulator raloxifene may be considered as an alternative in postmenopausal women with inactive lupus and without antiphospholipid antibodies and/or a history of thromboembolism [95].

Therapeutic use of DHEA is a treatment option for patients in whom therapy with bisphosphonates and raloxifene is contraindicated or is not tolerated. Low serum DHEAS levels, which are associated with low BMD, have been demonstrated in patients with SLE and offer the opportunity for the therapeutic use of DHEA to prevent bone loss. Studies in postmenopausal and GC-treated SLE patients with active disease have shown that DHEA treatment offers mild protection against bone loss [96]. However, the therapeutic use of this hormonal supplement is limited since DHEA is not approved for the prevention of bone loss in GC-treated SLE patients and is only available as a supplement in the United States and Europe.

Recent studies on the molecular pathways underlying bone metabolism have identified potential new therapeutic options for the prevention and treatment of osteoporosis, e.g. monoclonal antibodies against sclerostin. These agents may offer attractive therapeutic options for the management of osteoporosis in lupus patients in future.

### Guidelines for Screening and Treatment of Osteoporosis in SLE

Currently, no clear guidelines for screening and treatment of osteoporosis and the prevention of fractures in patients with SLE are available. In 2009, a quality indicator set for osteoporosis prevention and treatment in SLE was published, recommending screening for osteoporosis by BMD measurement in GC-treated patients [97]. The 2010 European League Against Rheumatism recommendations [74] advocate screening and follow-up for osteoporosis according to guidelines for postmenopausal women or patients on GCs, next to advices for adequate calcium and vitamin D intake, and lifestyle measures. However, in line with the 2010 American College of Rheumatology recommendations for prevention and treatment of GC-induced osteoporosis [75] and the National Osteoporosis Foundation [62] and the National Osteoporosis Guideline Group [63] guidelines, additional vertebral fracture assessment [using X-rays of the spine or Vertebral Fracture Assessment (VFA)] must be considered, since vertebral fractures occur frequently in patients with SLE and the detection of one or more prevalent vertebral fractures in patients with a BMD in the osteopenic range is an indication for initiation of anti-osteoporosis agents [62, 63]. The recently published 2017 American College of Rheumatology Guideline for the prevention and treatment of GC-induced osteoporosis [98] recommends assessment and reassessment of fracture risk in patients beginning or continuing long-term GC treatment, using history of osteoporotic fractures, BMD and 10-year risk of major osteoporotic fracture and hip fracture (FRAX) to estimate fracture risk in adults aged 40 years and older, and using history of osteoporotic fracture and BMD to estimate fracture risk in children and adults aged <40 years. However, using the FRAX tool to calculate fracture risk in patients with SLE has disadvantages as mentioned before. Moreover, osteoporosis screening and fracture risk assessment should also be considered in subgroups of patients not using GCs, i.e. postmenopausal women, men and patients with other risk factors for osteoporosis.

## Conclusion

In recent years, bone loss and fractures have been recognized as important disease complications in patients with SLE. Recent studies have provided important new insight in the epidemiology, the multifactorial aetiology and the clinical impact and economic burden of osteoporosis and fractures in this relatively rare disease. Population-based studies have demonstrated a 2.53-fold increased occurrence of osteoporosis and a 1.2–4.7-fold increased risk for symptomatic fractures in patients with SLE compared to matched controls. In addition, a high prevalence of morphometric vertebral deformities has been demonstrated in these relatively young patients and 1 in 3 of these patients have normal BMD, which finding points to the limited value of BMD measurement in the assessment of fracture risk and the multifactorial cause of fractures in SLE.

The aetiology of bone loss in SLE is multifactorial, including clinical osteoporosis risk factors, systemic inflammation, serological factors, metabolic factors, hormonal factors, possibly genetic factors and medication-induced adverse effects. In particular GC use has been recognized as an important factor contributing to the development of osteoporosis and fractures in SLE. Lupus patients are frequently treated with GCs, while GC use itself has been associated with an increased risk of fractures, independent of BMD. Therefore, the prescription of steroid-sparing agents is of major importance to minimize dose and duration of GC therapy in SLE patients with chronic active disease or with frequent disease flares, who are frequently or chronically treated with GCs. In addition, therapy with antimalarials and immunosuppressive agents is highly important to reduce inflammation in SLE, since the disease itself has been associated with reduced bone strength and high flare rate was reported as an independent risk factor for bone loss in SLE.

Advices for adequate calcium and vitamin D intake and lifestyle measures are important for all lupus patients, but are of major importance for women with SLE of childbearing age who have not completed their families yet, because limited drugs are available for the prevention and treatment of osteoporosis in this subgroup of patients.

Screening for vertebral fractures in addition to BMD measurement in the assessment of fracture risk in SLE is necessary, since these fractures occur frequently, and in the majority of cases asymptomatically, but have major clinical and often therapeutical consequences.

Future studies should focus on fall risk and its determinants and the relationship with the occurrence of fractures, since fall risk in patients with SLE might be increased, but no data on this subject are currently available. In addition, the recently reported high prevalence of frailty in 20% of the SLE patients warrants further studies on the influence of this potential additional risk factor for the increased occurrence of fractures in SLE.
